# Analysis of Intestinal Microflora and Metabolites From Mice With DSS-Induced IBD Treated With *Schistosoma* Soluble Egg Antigen

**DOI:** 10.3389/fcell.2021.777218

**Published:** 2021-11-09

**Authors:** Tianyu Zhu, Qingkai Xue, Yiyun Liu, Yongliang Xu, Chunrong Xiong, Jin Lu, Haitao Yang, Quan Zhang, Yuzheng Huang

**Affiliations:** ^1^ National Health Commission Key Laboratory of Parasitic Disease Control and Prevention, Jiangsu Provincial Key Laboratory on Parasite and Vector Control Technology, Jiangsu Institute of Parasitic Diseases, Wuxi, China; ^2^ Center for Global Health, School of Public Health, Nanjing Medical University, Nanjing, China; ^3^ Institute of Comparative Medicine, College of Veterinary Medicine, Yangzhou University, Yangzhou, China; ^4^ Jiangsu Co-innovation Center for Prevention and Control of Important Animal Infectious Diseases and Zoonoses, Yangzhou University, Yangzhou, China

**Keywords:** flora, inflammatory bowel disease, metabolism, schistosoma soluble egg antigen, dextran sulfate sodium salt (DXT)

## Abstract

**Objective:** This study aimed to analyze the changes in intestinal flora and metabolites in the intestinal contents of mice with inflammatory bowel disease (IBD) to preliminarily clarify the mechanism of action of *Schistosoma* soluble egg antigen (SEA) on IBD, thus, laying a research foundation for the subsequent treatment of IBD.

**Methods:** A total of 40 Institute of Cancer Research (ICR) mice were divided into four groups: control, SEA 50 μg, dextran sulfate sodium salt (DSS), and SEA 50 μg + DSS. The overall state of the animals was observed continuously during modeling. The colonic length was measured after 10 days of modeling. The degree of colonic inflammation was observed by hematoxylin and eosin staining. 16srRNA and liquid chromatography–mass spectrometry sequencing techniques were used to determine the abundance of bacteria and metabolites in the intestinal contents of mice in the DSS and SEA 50 μg + DSS groups, and the differences were further analyzed.

**Results:** After SEA intervention, the disease activity index score of mice with IBD decreased and the colon shortening was reduced. Microscopically, the lymphocyte aggregation, glandular atrophy, goblet cell disappearance, and colonic inflammation were less in the SEA 50 μg + DSS group than in the DSS group (*p* < 0.0001). After SEA intervention, the abundance of beneficial bacteria *prevotellaceae_UCG-001* was upregulated, while the abundance of the harmful bacteria *Helicobacter*, *Lachnoclostridium*, and *Enterococcus* was downregulated in the intestinal tract of mice with IBD. The intestinal metabolite analysis showed that SEA intervention decreased the intestinal contents of glycerophospholipids (lysophosphatidylcholine, lysophosphatidylethanolamine, phatidylcholine, and phatidylethanolamine) and carboxylic acids (L-alloisoleucine and L-glutamate), whereas increased bile acids and their derivatives (3B,7A,12a-trihydroxy-5A-cholanoic acid and 3A,4B, 12a-trihydroxy-5b-cholanoic acid). Combined microbiota–metabolite analysis revealed a correlation between these differential microbiota and differential metabolites. At the same time, the changes in the contents of metabolites and differential metabolites in the two groups also correlated with the abundance of the gut microbiome.

**Conclusions:** The study showed that SEA reduced DSS-induced inflammation in IBD and improved the symptoms of IBD in mice through the combined regulation of intestinal flora and intestinal metabolism. It suggested a potential possibility for the use of SEA in treating and regulating intestinal flora and metabolism in patients with IBD.

## Introduction

Inflammatory bowel disease (IBD) is a chronic, recurrent inflammatory bowel disease characterized by abdominal pain, diarrhea, rectal bleeding, and weight loss (Kaplan, 2015).In recent decades, the incidence of IBD has increased globally, and to date, it has affected millions of people and caused huge economic losses (Ben-Ami Shor et al., 2013). Currently, IBD therapy relies on frequent high doses of 5-aminosalicylic acid, corticosteroids, immunomodulators, and anti-tumor necrosis factor-α monoclonal antibodies (Damião et al., 2019; Jeong et al., 2019). However, these therapies are effective only in the early stage of IBD and relieve only the inflammatory symptoms of IBD, often with certain side effects and limitations, including immunosuppression, drug resistance, and huge costs (Mao and Hu, 2016). These drawbacks pose challenges to the treatment of IBD.

Although the pathogenesis of IBD remains to be further explored, most studies believe that IBD is related to immune imbalance (Nanini et al., 2018; Neurath, 2019; Mitsialis et al., 2020). As a common chronic parasitic worm (Huang et al., 2016a; Huang et al., 2016b; Deol et al., 2019), a schistosome develops a variety of mechanisms to manipulate the adaptive immune system of the host while infecting the host (Huang et al., 2016a; Huang et al., 2019; Huang et al., 2020a; Huang et al., 2020b; Buck et al., 2020; Zheng et al., 2020). Studies have shown that the host immune system gradually shifts from its own invasive T helper 1 (Th1) cell response to anti-inflammatory Th2 cell response 4–6 weeks after cercariae penetrated the skin of the host (Dunne and Cooke, 2005). In recent years, the use of *Schistosoma* soluble egg antigen (SEA) or its derivatives for autoimmune diseases is not uncommon (Driss et al., 2016; Li et al., 2020). It has been suggested that SEA can reduce the intestinal inflammatory symptoms of IBD and reduce the susceptibility to colitis (Floudas et al., 2019; Cleenewerk et al., 2020). However, the specific mechanism of action needs further exploration.

A growing body of evidence highlights the complexity, importance, and interactions between symbiotic bacteria and the host immune system in health and disease (Goto et al., 2015; Zhou et al., 2020). IBD is closely associated with changes in intestinal microbiota diversity and the disruption of the balance between symbiotic microbiota and potentially pathogenic microbiota components (Ni et al., 2017). Metabolites act as a bridge between the intestinal microbiome and the host (Dong et al., 2019; Lavelle and Sokol, 2020). Metabolites are small molecules produced as intermediates or end products of microbial metabolism, which transmit signals of intestinal microorganisms and affect immune maturation, immune homeostasis, host energy metabolism, and maintenance of mucosal integrity (Wilson and Nicholson, 2017). In addition, studies have shown that the metabolic profile of patients with IBD is different from that of normal people (Franzosa et al., 2019). Specific types of metabolites, especially bile acids, short-chain fatty acids, and tryptophan metabolites, are associated with the pathogenesis of IBD (Lavelle and Sokol, 2020). Therefore, this study preliminarily explored the mechanism of action of SEA on IBD based on the changes in intestinal flora and metabolites of the intestinal contents of mice with IBD and laid a research foundation for the subsequent clinical application of SEA and the treatment of IBD.

## Materials and Methods

### Materials

#### Experimental Animals

A total of 40 female ICR mice (age 6 weeks, weighing 26.22 ± 1.12 g) were purchased from SiPeiFe (Beijing) Biotechnology Co., Ltd (License Number: SCXK (Beijing) 2019–0,010, Quality Certificate Number: No: 110,324,201,104,129,683, Ethical review number: JIPD—2020–009).

#### Preparation of SEA

*Schistosoma japonicum* (Jiangsu strain) was preserved by the Jiangsu Institute of Parasitic Diseases, and the cercariae were escaped from infected Oncomelania snail in our laboratory and collected for animal experiments. New Zealand rabbits were infected with 1,500 cercariae each, and the *Schistosoma* eggs were collected from the liver and mesenteric venous plexus after 42 days of raising in the laboratory. Eggs were then mixed with 0.9% sodium chloride solution and ground for 20–30 min. After grinding, the mixture was centrifuged at 10,000 g for 10 min, and the supernatant was collected. The process was repeated three times, and the supernatant was filtered with a 0.22-μm pore size filter membrane. The concentration of crude protein was determined using the NanoDrop absorbance value and stored at –80°C for later use.

### Methods

#### Animal

A total of 40 mice were divided into four groups: SEA 50 μg (S), dextran sulfate sodium salt (DSS) (D), SEA50 μg + DSS (S + D), and control (C).

On day 0, the mice in the D and S + D groups were given 3% DSS (Sigma, lot#BCCD1174, Denmark) instead of pure water. On day 0 of DSS modeling, each mouse in the S and S + D groups was intraperitoneally injected with one dose of 50 μg SEA.The mice were euthanized after 10 days.

#### Collection of Intestinal Contents

For model making, the colon of each mouse was taken out after 10-days execution. The colonic contents were collected and placed in 1.5-ml cryogenic vials. The intestinal contents were first placed under liquid nitrogen for 10 min, immediately transferred to –80°C for subsequent detection.

#### Intestinal Histology

The length of the colon was measured, and a 1-cm sample from the distal colon was fixed in a 10% neutral buffer formalin. After fixation, the samples were fixed in paraffin, sectioned (5-µm thick), and stained with hematoxylin and eosin (HE). The histopathological changes were observed and recorded under a microscope.

The histological score of the colon was determined in a blinded manner. Cell infiltration: score 0, occasional inflammatory cells in the lamina propria (LP); 1, increased lymphocyte infiltration mainly at the base of the crypt; 2, inflammatory infiltration extending to the confluence of the mucosa; and 3, infiltration and extension through the wall. Tissue injury: score 0, no mucosal injury; 1, some areas (up to 50%) of crypts lost; 2, large-area crypt partial or total loss of 50–100%, and epithelial integrity; and 3, complete loss of large-area crypts and loss of epithelium.

#### Disease Activity Index Score

The disease activity index (DAI) of mice was calculated on 0, 3, 5, 7, and 10 days after modeling.

DAI was calculated for each mouse based on three parameters (body weight, stool shape, and stool bleeding), with a score of 1–4 for each parameter and a maximum cumulative DAI score of 12. The score was assigned as follows: 0, no weight loss, normal stool, and no blood; 1, weight loss 1%–3%; 2, weight loss 3–6%, stool thinning, and occult blood positive; 3, weight loss 6–9%; and 4, >9% weight loss, diarrhea, and overt bleeding.

#### Intestinal Flora Detection

Intestinal flora was detected using 16srRNA technology by specific methods as follows.

Bacterial DNA was isolated from the intestinal contents using MagPure Soil DNA LQ Kit (Magen, Guangdong, China), and the concentration of DNA was detected using agarose gel electrophoresis and a NanoDrop 2000 spectrophotometer (Thermo Fisher Scientific, MA, United States). Polymerase chain reaction (PCR) amplification of the V3-V4 hypervariable regions of the bacterial 16S rRNA gene was carried out in a 25 μl reaction using universal primer pairs (343F: 5′-TACGGRAGGCAGCAG-3′; 798R: 5′-AGG​GTA​TCT​AAT​CCT-3′).

The PCR products were purified with Agencourt AMPure XP beads (Beckman Coulter Co., United States). After purification, the PCR products were used as a template for second-round PCR amplification. The Amplicon quality was visualized using gel electrophoresis and quantified using a Qubit dsDNA assay kit.

The concentrations were then adjusted for sequencing. Sequencing was performed on an Illumina NovaSeq6000 system with two paired-end read cycles of 250 bases each (Illumina Inc., CA, United States; OE Biotech Company; Shanghai, China).

Vsearch software was used after the sequencing data were preprocessed to generate high-quality sequences. The sequences were grouped into multiple operational taxonomic units (OTUs) based on their similarity. The parameters for sequence similarity greater than or equal to 97% were classified as an OTU. QIIME software package (version 1.8.0) was used to select the representative sequences of each OTU, and all representative sequences were compared with the database for annotation. All representative reads were annotated and blasted against the SILVA database (v123) using the RDP classifier (v2.2). Alpha- and beta-diversity indexes were calculated using QIIME as previously described. To visualize diversity, we employed QIIME software to calculate estimators for each sample including the Shannon index, Chao1 index, Simpson’s Diversity Index, and Observed Species. In addition, β diversity between the communities was assessed by weighted UniFrac for principal coordinate analysis (PCoA).

Comparison of OTUs and taxonomy abundances was calculated using the Kruskal–Wallis test or Mann−Whitney analysis. Following statistical analyses with multiple comparisons, *p* values were corrected using the Benjamini–Hochberg method to control the false discovery rate (FDR). The resultant *p* values were FDR corrected with a significance threshold of 5%. Furthermore, the linear discriminant analysis (LDA) and the LDA effect size (LEfSe) measurements were used to find unique bacterial taxa among different groups LDA >2, FDR-p < 0.05 and *p* values <0.05 were considered statistically significant.

#### Intestinal Metabolite Analysis

Intestinal metabolites were detected by liquid chromatography–mass spectrometry (LC-MS) sequencing, and the specific methods were as follows.

For sample pretreatment, 60 mg of frozen intestinal contents were removed, put into a 1.5-ml Eppendorf tube, and mixed with 20 μl of internal standard (L-2-chlorophenylalanine, 0.3 mg/ml; methanol) and 600 μl of methanol–water (v:v = 4:1). Then, two small steel balls were added, placed in the refrigerator at –20°C for 5 min, and then ground in a grinder (60 Hz, 2 min). The mixture was subjected to ultrasonic extraction in an ice water bath for 10 min, allowed to stand for at –20°C for 30 min, and centrifuged for 10 min (13,000 rpm, 4°C). Then, 200 μl of the supernatant was put into an LC-MS vial and dried. The supernatant was mixed with 300 μl of methanol–water (v:v = 1:4) (eddy 30 s, ultrasonic 3 min), kept at –20°C for 2 h, and centrifuged for 10 min (13,000 rpm, 4°C). Subsequently, 150 μL of the supernatant was extracted with a syringe, filtered with a 0.22-μm organic-phase pinhole filter, transferred to an LC vial, and stored at –80°C until LC-MS analysis.

Quality control samples (QC) were prepared by mixing the extract of all samples in equal volume. The volume of QC was the same as that of samples, and LC-MS full-scan detection was performed. Data processing software Progenesis QI V2.3 was used to carry out qualitative and relative quantitative analyses of the original data, and the original data were preprocessed in a standardized way. The analytical instrument was a Dionex U3000 UHPLC (ultra-high-performance liquid chromatography system) in series with a QE Plus high-resolution mass spectrometer.

#### Statistical Analysis

SPSS 19.0 software was used to process the data. Analysis of variance was used for multi-group statistical analysis, and Dunnett’s multiple comparison was used to compare the differences between the groups. The *t* test was used for comparison between the two groups. A *p* value <0.05 indicates a statistically significant difference (∗*p* < 0.05, ∗∗*p* < 0.01, ∗∗∗*p* < 0.001, ∗∗∗∗*p* < 0.0001).

Analysis of microbiome and metabolome: Pearson correlation coefficients were calculated for microbiome and metabolome data integration. Based on the differential microorganism and metabolite expression, Pearson correlation coefficients were calculated by R; then, cluster analysis heat maps were drawn. The relationships between microorganisms and metabolites were visualized and interpreted using Cytoscape (version 3.4.0) with MetScape plug-in (version 3.1.3).

## Results and Analysis

### Changes in Disease Activity Index in Mice With Dextran Sulfate Sodium Salt-Induced Bowel Disease After SEA Intervention

**FIGURE 1:**
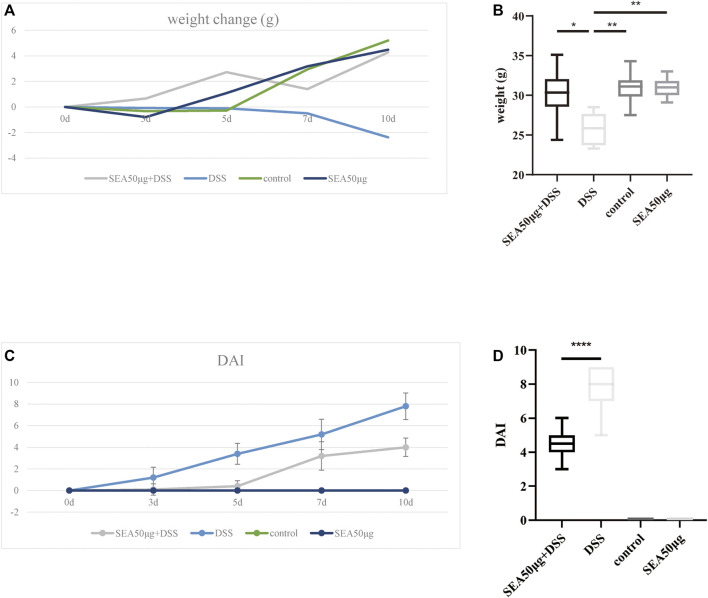
SEA downregulated the DAI score in mice with DSS-induced IBD. **(A)** Changes in average body weight in the control group, SEA 50 μg group, DSS group, and DSS + SEA 50 μg group after 0, 3, 5, 7, and 10 days. **(B)** Body weight in the control group, SEA 50 μg group, DSS group, and DSS + SEA 50 μg group after 10 days. **(C)** DAI changes in mice in the control group, SEA 50 μg group, DSS group, and DSS + SEA 50 μg group after 0, 3, 5, 7, and 10 days. **(D)** Comparison of the 10-days DAI score between the SEA 50 μg + DSS and DSS groups.

After DSS administration, the mice in the model group (D group) lost weight over time, while the weight in the other three groups showed an upward trend without significant differences. On the 10th day of administration, the average weight in the D group reduced by 2.33 g and was significantly lower than that in the other three groups (*p* < 0.001) (Figures 1A,B). Comprehensive DAI evaluation showed that the DAI score in the D and S + D groups increased with time, the mice lost weight, and diarrhea and fecal bleeding were increasingly aggravated. However, the increase in the DAI score in the S + D group slowed down, with a statistically significant difference compared with the D group (*p* < 0.0001) (Figures 1C,D), indicating that the intervention of SEA alleviated the progressive aggravation of enteritis in the D group.

### Effects of SEA on Dextran Sulfate Sodium Salt-Induced Induced Bowel Disease in Mice

**FIGURE 2:**
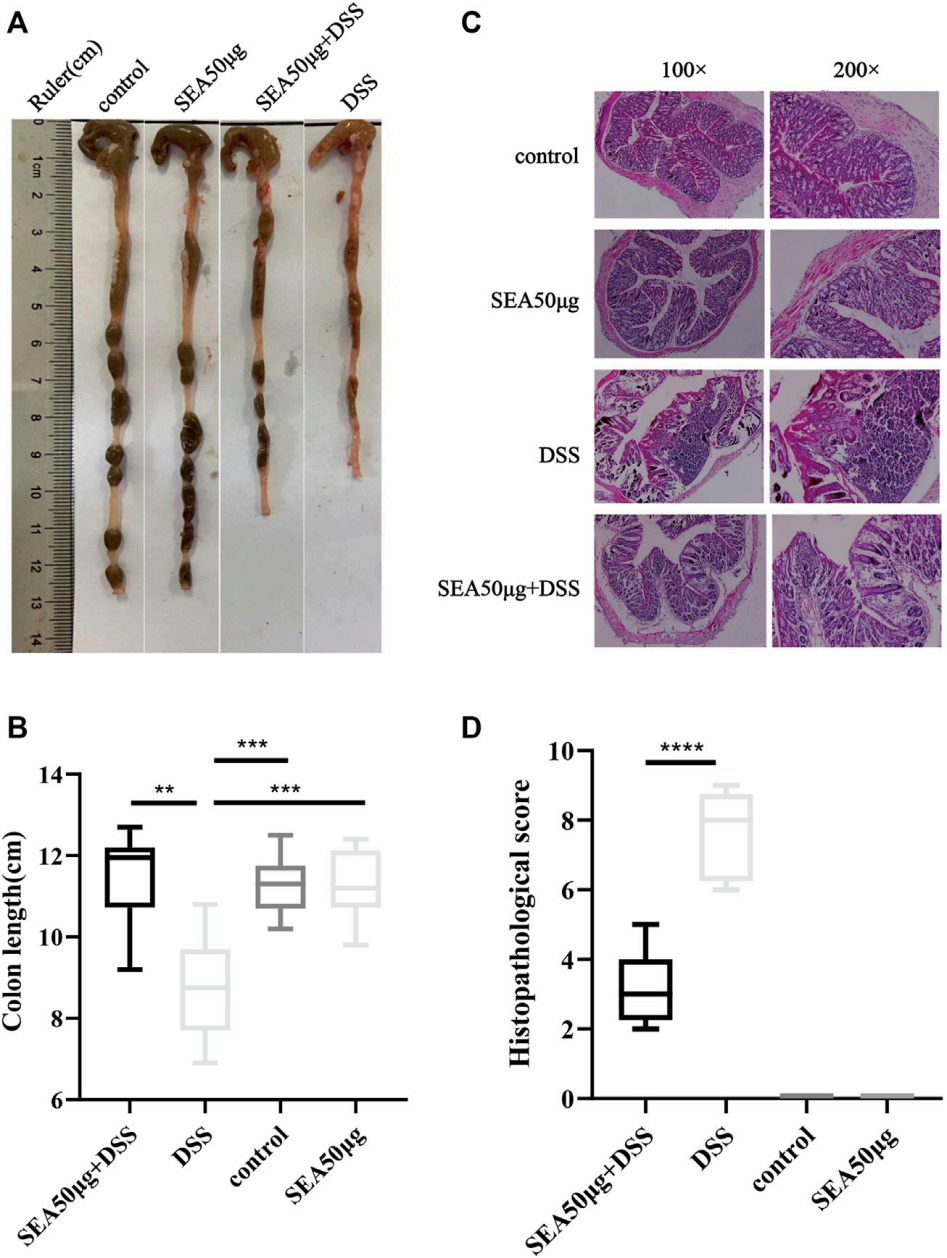
SEA improved DSS-induced colonic symptoms in IBD **(A)** Colonic status of mice in the control group, SEA 50 μg group, DSS group, and DSS + SEA 50 μg group after 10 days of subdivision. **(B)** Colonic tissue of mice was observed under the microscope after HE staining. **(C)** Comparison of colonic length on the 10th day in the control group, SEA 50 μg group, DSS group, and DSS + SEA 50 μg group **(D)** Histological scores of colon sections in the control group, SEA 50 μg group, DSS group, and DSS + SEA 50 μg group.

The results of colon status in the four groups showed that mucosal bleeding and fecal deformity occurred in the colon, and the colon length was significantly shortened in the D group compared with the other three groups (*p* < 0.001) (Figures 2A,B). After SEA intervention, the colon shortening was reduced (*p* < 0.001) (Figure 2B). Microscopically, lymphocyte aggregation, glandular atrophy, and goblet cell disappearance were observed in the colon of mice in the D group, indicating that the intake of DSS led to intestinal inflammation in mice and the development of significant colitis. After SEA intervention, the colon tissue of mice had less lymphocyte aggregation, glandular atrophy, goblet cell disappearance (*p* < 0.0001), and colonic inflammation (Figures 2C,D).

### Changes in the Intestinal Microflora in Mice With Dextran Sulfate Sodium Salt-Induced Induced Bowel Disease Treated With SEA Intervention

**FIGURE 3:**
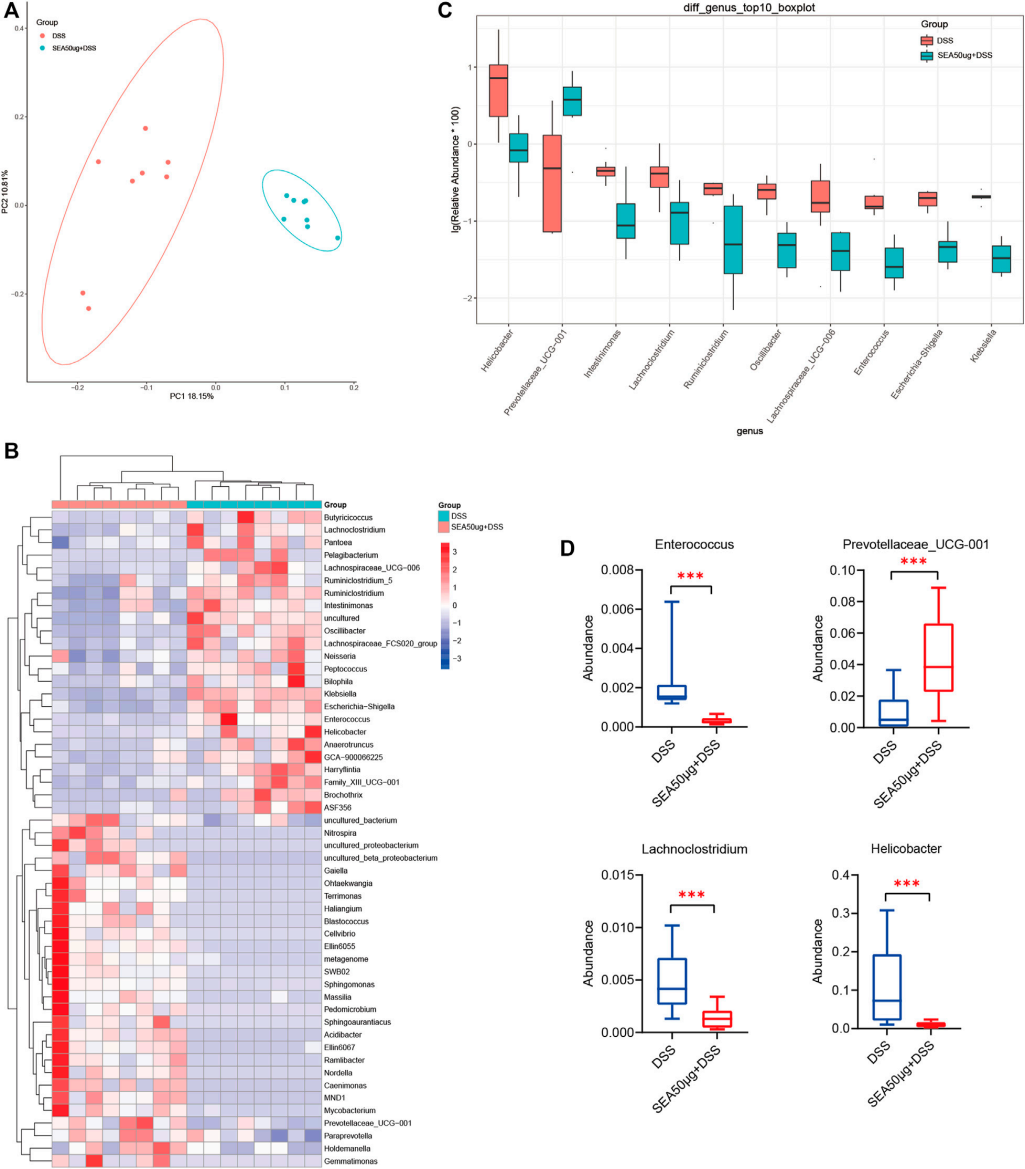
Intestinal microflora of mice with DSS-induced IBD mice treated with SEA. **(A)** Principal coordinate analysis (PCoA) of intestinal microflora in the DSS and SEA 50 μg + DSS groups (C50 group). Red represents DSS group, blue represents SEA 50 μg + DSS group, and each icon represents a group of samples. **(B)** DSS and SEA 50 μg + DSS group showed significantly differences after *t* test analysis. Comparative heat map (at the genus level). Each column represents a sample, and orange represents the SEA 50 μg + DSS group. **(C)** Comparison of the abundance of the first 10 species in the DSS and SEA 50 μg + DSS group (at the genus level) **(D)** Comparison of the abundance of intestinal *Helicobacter, Lachnoclostridium, Enterococcus,* Prevotellaceae*_UCG-001* in the DSS and SEA 50 μg + DSS group (at the genus level).

This study further analyzed the changes in intestinal microflora in the S + D and D groups to explore the protective mechanism of SEA on DSS-induced IBD. PCoA (Figure 3A) showed that the samples between the two groups were concentrated and no overlap occurred between the two groups, indicating that the samples of each group were well represented and comparable.

Subsequently, we selected the top 10 differential microflora in the *t* test analysis (Figure 3B). Four kinds of bacteria were found to be associated with enteritis, including *Helicobacter* (*p* < 0.05), *Lachnoclostridium* (*p* < 0.01), Prevotellaceae*_UCG-001* (*p* < 0.01), and *Enterococcus* (*p* < 0.01) (Figure 3C). The abundance of harmful bacteria, such as *Helicobacter*, *Lachnoclostridium*, and *Enterococcus*, was downregulated (Castaño-Rodríguez et al., 2017; Ben Braïek and Smaoui, 2019; Cao et al., 2020a; Cao et al., 2020b; Liang et al., 2020), while the abundance of beneficial bacteria, such as Prevotellaceae*_UCG-001* (Cignarella et al., 2018), was upregulated in the S + D group compared with the D group (Figure 3D). These results suggested that the changes in SEA-induced microflora might be a mechanism of action of SEA to protect intestinal inflammation; the intestinal microflora of mice with DSS-induced IBD was improved by upregulating the abundance of beneficial bacteria and downregulating the abundance of harmful bacteria.

### Changes in Intestinal Content Metabolism in Mice With Acute DSS-Induced Dextran Sulfate Sodium Salt Treated With SEA Intervention

**FIGURE 4:**
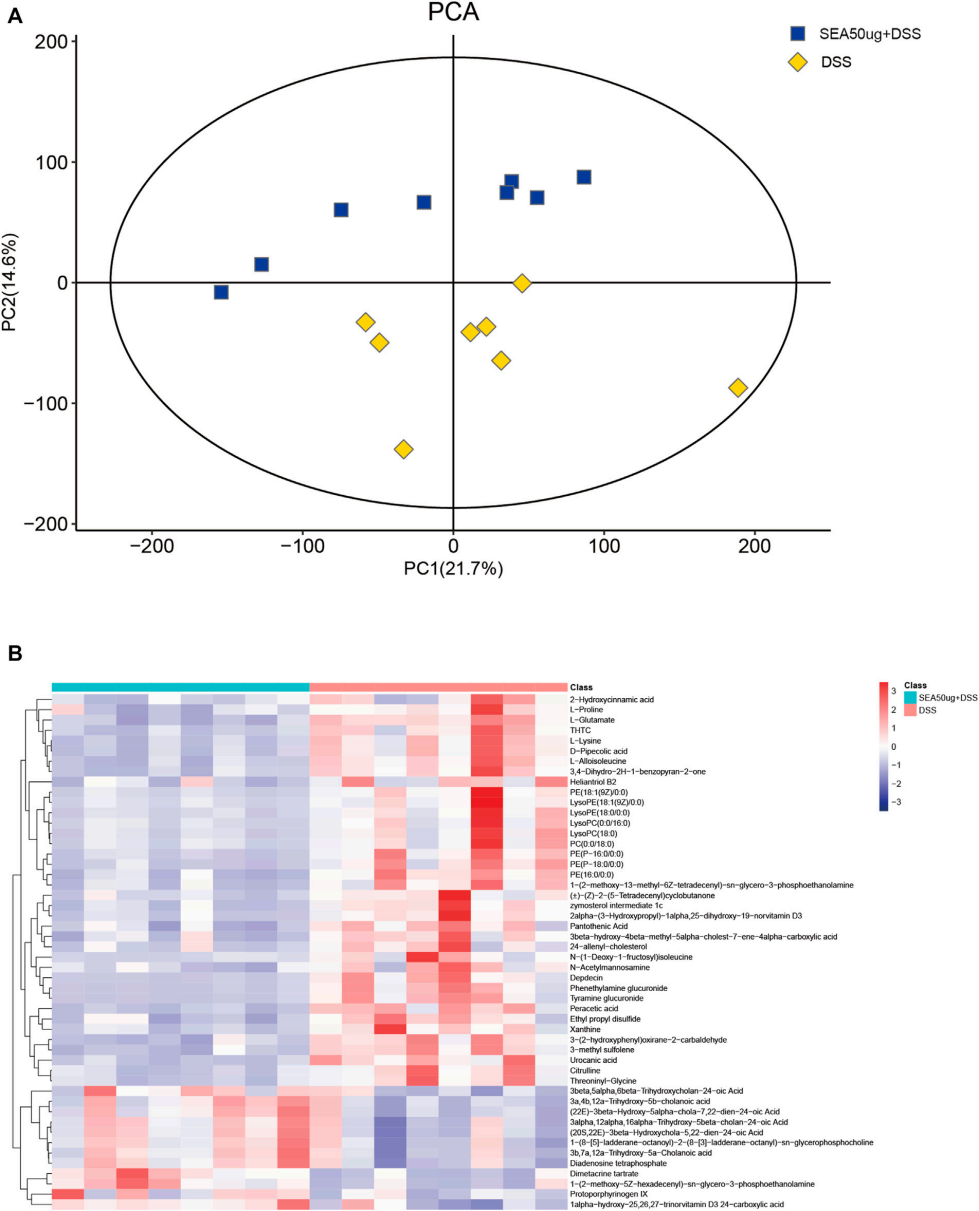
SEA altered the contents of DSS-induced intestinal metabolites in mice with IBD. **(A)** 2D principal component analysis (PCA) of the contents of intestinal metabolites in the DSS and SEA 50 μg + DSS groups. Yellow represents the DSS group, and blue represents the SEA 50 μg + DSS group. Each icon represents a set of samples. **(B)** Heat maps of the top 50 differential metabolites of intestinal contents in the DSS and SEA 50 μg + DSS groups. The left blue bar represents the DSS group, and the right side represents the SEA 50μ g + DSS group. Each small square in the figure corresponds to a mass error. The red square represents a positive mass error, while the blue square represents a negative mass error. The darker the color, the greater the value.

According to 2D PCA (Figure 4A) of metabolites in the S + D and D groups, the samples in each group were concentrated and no overlap occurred between the groups, indicating good representativeness and comparability between the two groups.

The analysis of the top 50 metabolites (Figure 4B) between the two groups showed that glycerophospholipids [lysophosphatidylcholine (LPC), lysophosphatidylethanolamine (LPE), phatidylcholine (PC), and phatidylethanolamine (PE)] and carboxylic acids (l-alloisoleucine and L-glutamate) were downregulated, and those of bile acids and their derivatives (3B,7A,12a-trihydroxy-5A-cholanoic acid and 3A,4B, 12a-trihydroxy-5b-cholanoic acid) were upregulated in the intestinal content of mice with IBD after SEA intervention. These differences in metabolites might be related to the inflammatory protection mechanism of SEA in mice with IBD.

### Association Between Intestinal Content Metabolism and Metabolic Flora Changes in Mice

**FIGURE 5:**
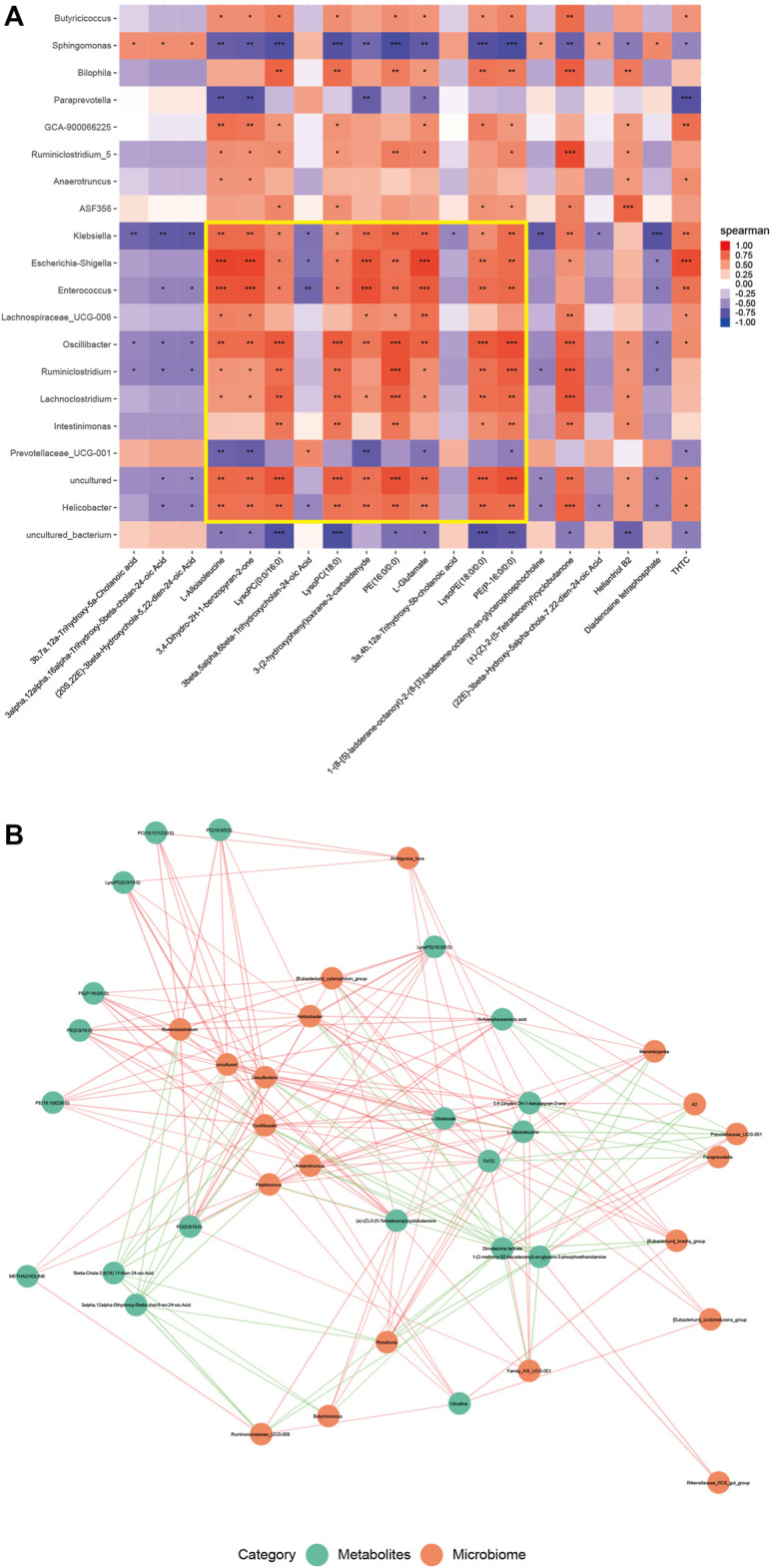
The contents of intestinal metabolites in mice correlated with the abundance of microflora. **(A)** Heat map of the first 20 different microflora and the first 20 different metabolites in the DSS and SEA 50 μg + DSS groups. The horizontal axis represents the differential metabolites, and the vertical axis represents the differential flora. Each small square represents a Pearson correlation coefficient, indicating the correlation between the corresponding metabolite and the bacterial community. The value is between –1 and 1. The closer the value to 0, the lower the correlation, and the closer the value to –1 or 1, the higher the correlation. Red represents a positive correlation, and blue a negative correlation; the darker the color, the stronger the correlation. The asterisk indicates the *p* value of the correlation coefficient, indicating whether the correlation is significant. **(B)** Correlation diagram of the first 20 different microflora and the first 20 different metabolites in the DSS and SEA 50 μg + DSS groups. Each green dot represents a differential metabolite, and each orange dot represents a differential flora. The distance between the two lines represents the size of their correlation, and the closer the line, the greater the correlation. In addition, different colors represent positive and negative correlations, with red representing positive values and green representing negative values.

A correlation between differential flora and differential metabolites was observed (Figure 5A), especially among the differential flora and differential metabolites related to enteritis (Figure 5A). Among these, the abundance of harmful bacteria *Helicobacter*, *Lachnoclostridium*, and *Enterococcus* positively correlated with the contents of glycerophospholipids, carboxylic acids, and their derivatives, and negatively correlated with the contents of bile acids and their derivatives. In contrast, the abundance of beneficial bacteria Prevotellaceae*_UCG-001* followed the opposite pattern (Figure 5B). This was also validated by the metabolic analysis results of the two groups.

Therefore, SEA may have a regulatory effect on intestinal flora and intestinal metabolites in mice with DSS-induced IBD, which is mainly manifested by improving the composition of intestinal flora, affecting the abundance of microbiota-related intestinal metabolites, and thus exerting a protective effect on DSS-induced IBD.

## Discussion

Parasitic worms can regulate the immune response and change the intestinal flora structure of the host, which has potential application prospects in treating autoimmune diseases (Bach, 2018; Castro Rocha et al., 2020).

In this study, the intraperitoneal injection of *Schistosoma japonicum* SEA was used to observe the efficacy of SEA against DSS-induced IBD. The results after SEA intervention showed that the body weight, DAI score, colon length, histological score of HE, and other indicators of the severity of enteritis were improved. These results indicated that the intestinal inflammation caused by DSS reduced after SEA intervention. IBD is closely related to the change in intestinal microbiota and the disruption of the balance between symbiotic microbiota (Weingarden and Vaughn, 2017). Many studies on IBD have described how changes in the composition and function of the microbiome are critical to the organisms (Larabi et al., 2020; Yang et al., 2021). The gut microbiome converts nutrients ingested into metabolites of the gut microbiome or host cells, making them act as informational messengers between the gut microbiome and host cells (Sittipo et al., 2019). Therefore, the composition of intestinal microbiota and its metabolites has a significant impact on the occurrence and development of IBD (Postler and Ghosh, 2017). In this study, 16srRNA gene sequencing and LC-MS sequencing were performed on the intestinal contents of mice in the D and S + D groups to observe the diversity, abundance, and changes in intestinal content metabolism in the two groups and to explore the correlation between intestinal flora, metabolism, and intestinal inflammation in mice with IBD after SEA intervention.

We found that SEA administration could downregulate the abundance of three specific enterica-related pathogenic bacteria, *Helicobacter*, *Lachnoclostridium*, and *Enterococcus*, and upregulate the abundance of beneficial intestinal bacteria Prevotellaceae*_UCG-001*. In a meta-analysis of environmental risk factors in clinical IBD samples, *Helicobacter* infection was identified as one of the nine factors that increased the risk of IBD (Castaño-Rodríguez et al., 2017; Piovani et al., 2019). *Lachnoclostridium* was significantly enriched in patients with colorectal cancer (Liang et al., 2020). *Enterococcus*, an opportunistic pathogen, is often associated with infection in clinic (Fiore et al., 2019). The lack of Prevotellaceae, a newly discovered flora in recent years, led to elevated levels of intestinal endotoxins and damage to the intestinal mucosal barrier (Cignarella et al., 2018). SEA administration significantly reduced the abundance of *Helicobacter*, *lachnoclostridium,* and *Enterococcus* in mice with IBD and upregulated the abundance of Prevotellaceae*_UCG-001*. These changes in enterica-related microflora further indicated that SEA played a protective role in DSS-induced IBD by improving the composition of intestinal microflora.

We also detected and analyzed the metabolic level of intestinal contents to explore further the changes in intestinal metabolites caused by intestinal flora. SEA intervention decreased the contents of glycerophospholipids (LPC, LPE, PC and PE) and carboxylic acids (l-alloisoleucine and L-glutamate) in the intestinal contents of mice with DSS-induced IBD. The contents of bile acids and their derivatives (3B,7A,12a-trihydroxy-5A-cholanoic acid and 3A,4B, 12a-trihydroxy-5b-cholanoic acid) were upregulated. Among these, bile acid and glutamate have been proved to be closely related to the pathogenesis of IBD (Sorrentino et al., 2020; Fiorucci et al., 2021). The release of intestinal bile acids may promote the regeneration of intestinal stem cells and epithelial cells, reducing the severe symptoms of IBD (Sorrentino et al., 2020). Glutamate receptors affect intestinal function (visceral sensitivity and motility) and brain function (stress response, mood, and behavior), and are involved in the pathogenesis of IBD (Baj et al., 2019). In addition, studies have shown that patients with IBD have significant changes in plasma lipid and metabolic profiles, most of which are the elevated contents of glycerophospholipids and linoleic acid (Tefas et al., 2020); IBD disorders are also implicated in the metabolism of glycerophospholipids in the body (Guan et al., 2020). SEA intervention changed the abundance of these IBD-related differential metabolites, suggesting that its anti-inflammatory mode of action was related to intestinal metabolites in mice with IBD.

Based on the bidirectional regulation of intestinal flora and intestinal metabolites in IBD, we conducted a joint analysis of intestinal differential metabolites and differential flora. The results showed correlations between the main differential microflora and the main differential metabolites. At the same time, the metabolite content and differential metabolite abundance in the two groups also correlated with the abundance of intestinal flora. After SEA administration, the content of differential metabolites having a negative correlation with the abundance of differential intestinal flora in intestinal contents increased, while the content of positively correlated differential metabolites decreased. On the one hand, this was verified by the results of intestinal flora and metabolic sequencing; on the other hand, it also indicated that the protective mechanism of SEA in DSS-induced IBD might play a role through the joint regulation of intestinal flora and intestinal metabolism. It mainly upregulated the abundance of beneficial intestinal bacteria and downregulated the abundance of harmful intestinal bacteria, so as to change the regulation mode of intestinal metabolic spectrum in mice with enteritis. In addition, we also found a group of bacteria such as *Sphingomonas*, although their relationships with enteritis have rarely been reported, which showed a strong correlation with these differential metabolites. Whether these bacteria also play a relevant role in the inflammatory inhibition of IBD is worth further exploration.

In conclusion, the results of this study proved that SEA protected the DSS-induced inflammatory response in IBD and improved the symptoms of IBD in mice through the joint regulation of intestinal flora and intestinal metabolism, thus proposing a potential possibility for the use of SEA in treating and regulating intestinal flora and metabolism in patients with IBD. Of course, the specific mechanism underlying the increase in the abundance of beneficial bacteria and the change in the immune function caused by SEA remains to be studied. Therefore, the clinical application of SEA needs further exploration.

## Data Availability

The datasets presented in this study can be found in online repositories. The names of the repository/repositories and accession number(s) can be found below: https://www.ncbi.nlm.nih.gov/, https://www.ebi.ac.uk/metabolights/.
